# Glial degeneration with oxidative damage drives neuronal demise in MPSII disease

**DOI:** 10.1038/cddis.2016.231

**Published:** 2016-08-11

**Authors:** Cristina Zalfa, Chiara Verpelli, Francesca D'Avanzo, Rosella Tomanin, Cinzia Vicidomini, Laura Cajola, Renzo Manara, Carlo Sala, Maurizio Scarpa, Angelo Luigi Vescovi, Lidia De Filippis

**Affiliations:** 1 Department of Biotechnology and Biosciences, University Milan Bicocca, Piazza della Scienza 2, Milano 20126, Italy; 2 CNR Neuroscience Institute and Department of Biotechnology and Translational Medicine, University of Milan, Via Vanvitelli 32, Milano 20129, Italy; 3 Laboratory of Diagnosis and Therapy of Lysosomal Disorders, Department of Women's and Children's Health, University of Padova, Via Giustiniani 3, Padova 35128, Italy; 4 Stemgen Srl, Viale Ca' Granda, Milano, Italy; 5 Department of Neuroradiology, University of Salerno, Via S Allende, Baronissi 84081, Italy; 6 Stem Cells Laboratory, Cell Factory and Biobank, Azienda Ospedaliera ‘Santa Maria', Viale Tristano da Joannuccio 1, Terni 05100, Italy; 7 Casa Sollievo della Sofferenza, Viale Cappuccini 2, San Giovanni Rotondo (FG) 71013, Italy

## Abstract

Mucopolysaccharidosis type II (MPSII) is a lysosomal storage disorder due to the deficit of the iduronate 2-sulfatase (IDS) enzyme, causing progressive neurodegeneration in patients. Neural stem cells (NSCs) derived from the IDS-ko mouse can recapitulate MPSII pathogenesis *in vitro*. In differentiating IDS-ko NSCs and in the aging IDS-ko mouse brain, glial degeneration precedes neuronal degeneration. Here we show that pure IDS-ko NSC-derived astrocytes are selectively able to drive neuronal degeneration when cocultured with healthy neurons. This phenotype suggests concurrent oxidative damage with metabolic dysfunction. Similar patterns were observed in murine IDS-ko animals and in human MPSII brains. Most importantly, the mutant phenotype of IDS-ko astrocytes was reversed by low oxygen conditions and treatment with vitamin E, which also reversed the toxic effect on cocultured neurons. Moreover, at very early stages of disease we detected *in vivo* the development of a neuroinflammatory background that precedes astroglial degeneration, thus suggesting a novel model of MPSII pathogenesis, with neuroinflammation preceding glial degeneration, which is finally followed by neuronal death. This hypothesis is also consistent with the progression of white matter abnormalities in MPSII patients. Our study represents a novel breakthrough in the elucidation of MPSII brain pathogenesis and suggests the antioxidant molecules as potential therapeutic tools to delay MPSII onset and progression.

Mucopolysaccharidosis type II (MPSII, Hunter Syndrome, MIM: 309900) is caused by mutations in the gene encoding the lysosomal enzyme iduronate 2-sulfatase (IDS), with resulting accumulation of the glycosaminoglycans (GAGs), heparan and dermatan sulfate in the lysosomes. MPSII may occur in attenuated or severe forms, the latter with strong and progressive neurological involvement. Treatment with enzyme replacement therapy (ERT) is partly effective in peripheral organs but insufficient to rescue the central nervous system (CNS) disease.^1^ The mechanisms involved in CNS impairment are still poorly understood. We recently showed that neural stem cells (NSCs) derived from the subventricular zone (SVZ) of the IDS-ko mouse, the animal model of MPSII, mimic brain pathogenesis *in vitro*. Based on our findings, we suggested the involvement of an anomalous NSC-derived glial progenitor differentiation pathway as a potential mechanism for MPSII neuropathology.^2^

Astrocytes, the major population of glial cells in the adult mammalian brain, are crucial for neuronal support, synaptic transmission and information processing.^3^ They maintain CNS homeostasis by regulating ion concentrations, by metabolizing neurotransmitters, and by controlling cerebral blood flow. Moreover, astrocytes vigorously react to brain damage or disease and have neurogenic properties in specific brain niches.^3^ Upregulation of the glial fibrillary acidic protein (GFAP) with astrogliosis is a hallmark of neuroinflammation in the diseased brain^4^ of both mouse model and Hunter patients^2^ and oxidative damage has been documented in Hunter patients.^5, 6^ We here investigate the development of the neuroinflammatory environment during MPSII progression in the aging IDS-ko mouse brain. Our work shows that microgliosis/astrogliosis, along with an increase of oxidative damage-related hallmarks, coincide with GAG accumulation as very early events in MPSII pathogenesis and precede glial degeneration, which finally leads to neuronal death. In this scenario, the exploitation of antioxidants and neurotrophins^2^ or the transplantation of NSC-derived glial progenitors combined with ERT may be considered for MPSII treatment.

## Results

### IDS deficit leads to a visible mutant phenotype in IDS-ko NSC-derived astrocytes

**Figure 1:**
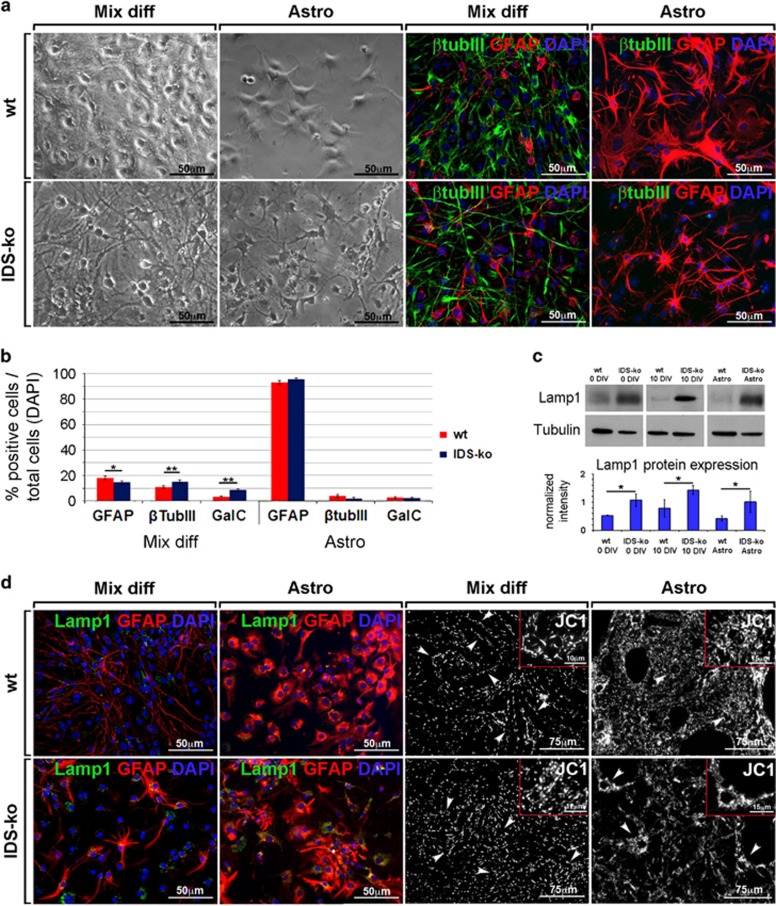

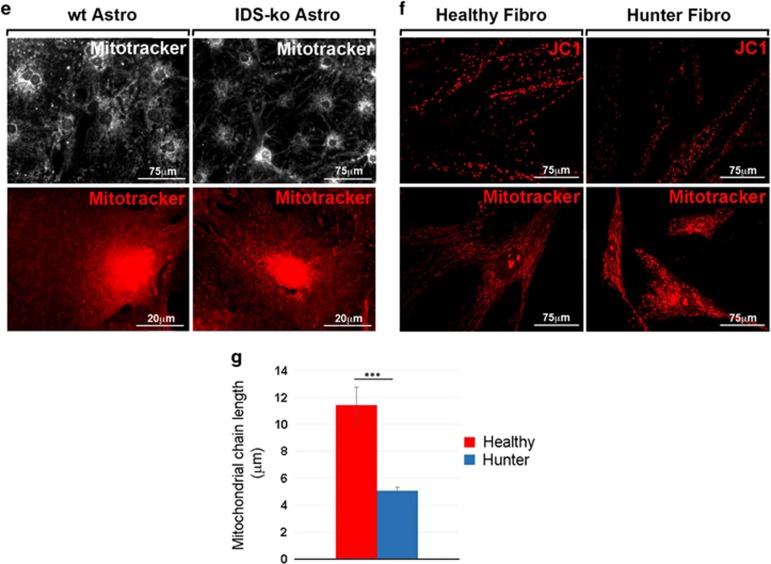
Characterization of IDS-ko NSC-derived astrocytes. (**a**) Wt and IDS-ko NSCs were differentiated to mixed progeny (Mix diff) for 10 div, or to pure astrocytes (Astro), through a specific ‘astrogenic' protocol for 21 div. Phase-contrast images show the different morphology of pure astrocyte cultures with respect to mixed differentiated progenies. Fluorescence microscopy images of wt and IDS-ko standard differentiated NSCs and Astro show the different neural cell types. Cells stained against *β*-tubIII with GFAP show the absence of *β*-tubIII+ cells in astrocyte cultures. Scale bars: 50 *μ*m. (**b**) Quantitative analysis of *β*-tubIII+, GFAP+ and GalC+ cells over total DAPI+ nuclei in mixed differentiated progeny and astrocytes deriving from wt and IDS-ko NSCs. (**c**) Western blot analysis of Lamp1 show that the amount of the lysosomal cargo is higher in mutant stem cells (0 div), mixed differentiated progeny (10 div) and Astro compared with wt. (**d**) Fluorescence microscopy images of wt and IDS-ko standard differentiated NSCs and astrocytes stained against Lamp1 with GFAP and JC1 show the enhanced lysosomal aggregation and impaired mitochondrial distribution in mutant astrocyte cultures compared either with mutant mixed progenies (white arrows) or to wt astrocytes. Scale bars: 50–75 *μ*m. Inset scale bar: 10–15 *μ*m. (**e**) Fluorescence (top) and confocal microscopy images (high magnification, bottom) of wt and IDS-ko astrocytes stained with MitoTracker. Scale bars: 75 *μ*m (top) and 20 *μ*m (bottom). (**f**) Images of human fibroblasts from Hunter patients and healthy controls stained with JC1 and MitoTracker show a more disorganized and fragmented mitochondrial structures in Hunter cells. Scale bars: 75 *μ*m. (**g**) Quantitative analysis of mitochondrial chain length in human fibroblasts showing a significant shorter mitochondrial chain length in Hunter versus healthy fibroblasts. *t*-Test for all experiments was applied. Data are reported as mean±S.E.M.

We previously showed that glial degeneration precedes neuronal demise both *in vivo*, in the MPSII mouse brain, and *in vitro*, in NSCs derived from the SVZ of adult IDS-ko mouse brain.^2^ To characterize astroglial degeneration in MPSII pathogenesis, we derived pure astrocytes from IDS-ko NSCs and analyzed their metabolic and neural differentiation patterns compared with astrocytes differentiated from control NSCs. Astroglial progenitors were enriched from IDS-ko NSC neurospheres according to a defined protocol (see Materials and Methods) and then terminally differentiated to mature astrocytes for 21 days *in vitro* (div) (Figures 1a and b). Wild-type (wt) syngenic NSC lines were used as control. Most cells were GFAP+ both in wt and in IDS-ko-differentiated progenies, whereas no *β*-tubulinIII+ or GalC+ cells were detectable in the astroglial cultures (Figures 1a and b). As a pathological marker, we evaluated the lysosomal size/number through western blot analysis (Figure 1c), and immunostaining (Figure 1d) of the Lamp1 (lysosomal-associated membrane protein 1) protein, a marker of late endosome and lysosome membrane.^7^ Lamp1 expression was higher in IDS-ko versus wt cells and enhanced with differentiation, with similar evidence in astrocytes derived from IDS-ko NSCs (Figure 1c). As the accumulation of intralysosomal material causes a reduction of mitochondrial turnover and activity,^8^ we tested the mitochondrial pattern in IDS-ko-differentiated cells and astrocytes both by MitoTracker and JC1 assay (Figures 1d and e) to stain, respectively, all or active mitochondria. A spotted and disorganized distribution of mitochondria was evident in IDS-ko in contrast with an extended linear network^9, 10^ in wt cells (Figure 1d). In particular, active mitochondria are heterogeneously aggregated in IDS-ko astrocytes, whereas homogeneously distributed in wt astrocytes (Figure 1d insets), suggesting that IDS deficit affects both lysosomal and mitochondrial distribution. Accordingly, our observation of human Hunter fibroblasts stained with MitoTracker and JC1 (Figure 1f) showed mitochondria spread in more fragmented structures and with a significantly shorter mitochondrial chain length compared with controls (Figure 1g), consistent with what observed in other lysosomal storage diseases (LSDs).^11, 12^

Moreover, Nissl staining of IDS-ko mouse brain showed effective neuronal degeneration only from the advanced symptomatic stage to the end stage (Supplementary Figure 1a), definitely prompting us to focus on the astroglial damage, in the study of the early phase of disease development and progression.

As lysosomal–mitochondrial cross-talk is important in the development and response to oxidative stress and as the formation of lipofuscin is representative of reactive oxygen species (ROS)-induced lysosomal damage,^8^ we tested the presence of lipofuscin aggregates *in vitro*, in differentiated NSCs and in pure astrocytes, as well as *in vivo*, in murine IDS-ko and in human Hunter brains. In differentiated NSCs, lipofuscin was already detectable in glial cells, but appeared strikingly evident in pure astrocytes after 21 div of maturation. Accordingly, lipofuscin was observed in mature areas of IDS-ko mouse brain, such as the cortex. Lipofuscin aggregates increased with age and reached a plateau from the symptomatic to the end stage of disease (Supplementary Figure 1b). No labeling was ever observed in the SVZ. Interestingly, a morphological analysis showed that lipofuscin appears earlier in astroglial cells and only at the end stage of the disease (11 months) in neuronal cells, thus mirroring the pattern of lysosomal alteration.^2^ Lipofuscin accumulation was also detected in a brain sample of a Hunter patient (Supplementary Figure 1c).

### Pathological phenotype in mutant astrocytes can be rescued by antioxidant treatment

To limit the oxidative stress by approximating *ex vivo* the physiological environment in the healthy brain,^13^ we differentiated IDS-ko NSCs into astrocytes under standard (16–20% O_2_) and low oxygen culture conditions (5% O_2_).

**Figure 2:**
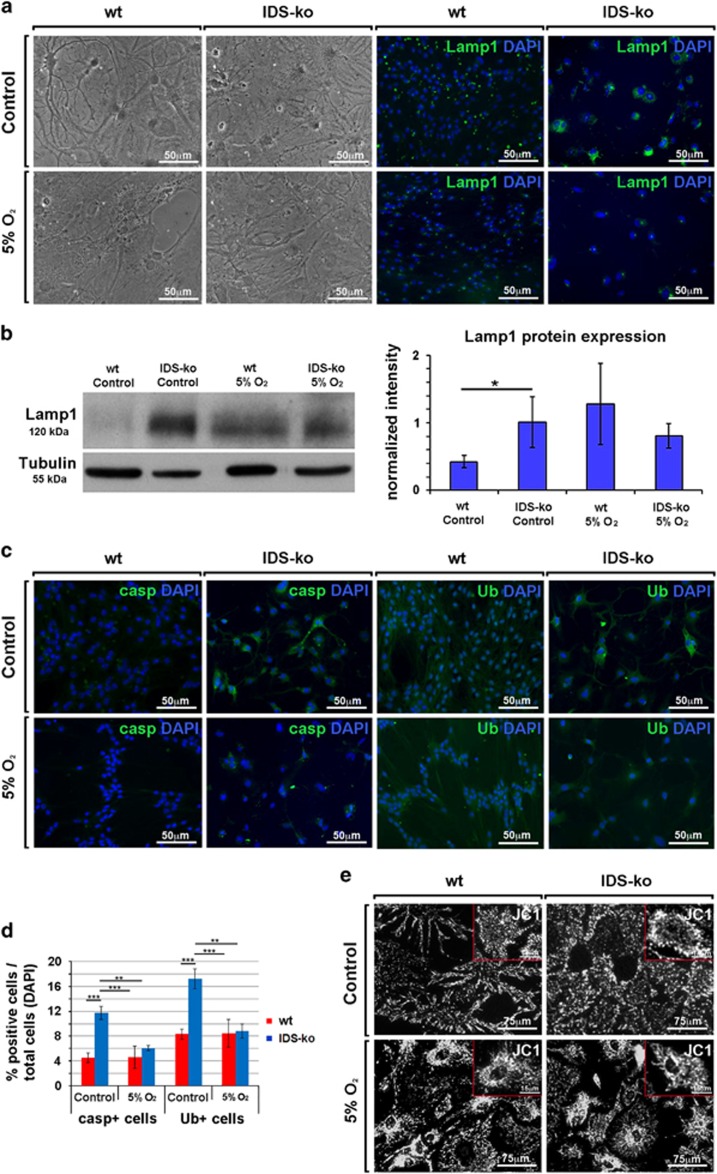
Effects of low oxygen on mutant astrocytes. (**a**) Wt and IDS-ko NSC-derived astrocytes were cultured for 21 div under standard (20% O_2_) or low (5% O_2_) oxygen culture condition. Phase-contrast images show the major spreading of the cell body and processes under low oxygen. Immunostaining with Abs against Lamp1 show the reduced number of lysosomal aggregates in mutant astrocytes by low oxygen compared with standard condition. Scale bars: 50 *μ*m. (**b**) Western blot analysis of Lamp1 expression in wt and IDS-ko NSC-derived astrocytes under standard (20% O_2_, Control) or low (5% O_2_) oxygen culture condition show that lysosomal amount in mutant cells is normalized by low oxygen. (**c**) Immunostaining with Abs against Ub and caspase-3 (casp) shows the reduced number of apoptotic cells in mutant astrocytes by low oxygen compared with standard condition. Scale bars: 50 *μ*m. (**d**) Quantitative analysis of caspase-3+ and Ub+ cells over the total DAPI+ nuclei under standard and low oxygen conditions. (**e**) JC1 *in vivo* assay shows a partial rescue of mitochondrial distribution in mutant astrocytes under low oxygen. Scale bars: 75 *μ*m. Inset scale bar: 13–15 *μ*m. *t*-Test for all experiments was applied. Data are reported as mean±S.E.M.

Mutant astrocytes displayed a morphology that resembled a normal phenotype under 5% O_2_ compared with standard conditions (Figure 2a). A parallel reduction of Lamp1 levels was observed either in mutant or in wt astrocytes (Figures 2a and b), with emphasized evidence in mutant cells, suggesting that low oxygen could partially rescue the pathological phenotype. Interesting, although not significant, we observed that Lamp1 expression in wt cells tended to increase at low oxygen, likely because of compensatory modulations of metabolism under different environmental conditions.^14, 15, 16^ We further investigated the effects of low oxygen conditions on apoptosis and mitochondrial status demonstrating a reduction of lipofuscin accumulation (Supplementary Figure 2c), ubiquitin (Ub) aggregates and caspase-3+ levels (Figures 2c and d) in mutant cells. Similarly, the JC1 assay showed in both wt and IDS-ko astrocytes an overall increase of the number of active mitochondria, with mutant cells displaying a wt-like reorganization of mitochondrial distribution (Figure 2e). We tested whether low oxygen conditions could be mimicked by antioxidant molecules. Treatment with vitamin E^17^ elicited results similar to those obtained with low oxygen (Supplementary Figures 2a–c), suggesting the use of antioxidant molecules as a possible strategy to reduce apoptosis and oxidative damage in MPSII.

### IDS-ko astrocytes drive neuronal death in coculture with primary cortical neurons

**Figure 3:**
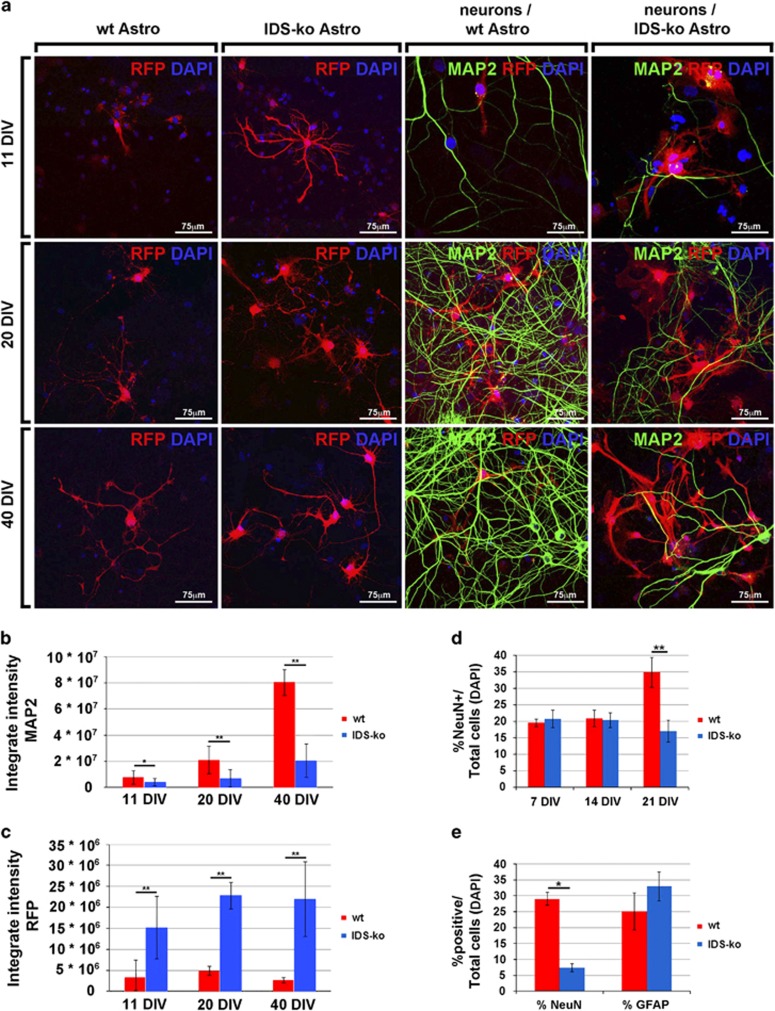
Coculture of cortical neurons with mature IDS-ko NSC-derived astrocytes. (**a**) Wt and IDS-ko NSC-derived mature astrocytes (21 div) were infected with lentiviral vector carrying the rfp reporter gene and cocultured for further 40 div with rat primary neurons. Confocal microscopy images showing the immunostaining of the neuronal marker MAP2 (green) and of RFP (red). Scale bars: 75 *μ*m. (**b** and **c**) Densitometric analysis of MAP2 and RFP intensity at 11, 20 and 40 div of coculture shows that a remarkable reduction of MAP2 neuronal expression (**b**) is accompanied by a massive increase of RFP intensity (**c**) where primary neurons are cocultured with mutant astrocytes. (**d**) Quantitative analysis of NeuN+ cells in cocultures of primary neurons with NSC-derived astrocytes previously differentiated for 7, 14 and 21 div. The reduction of NeuN+ cells over total DAPI+ nuclei is evident only with mature astrocytes (21 div). (**e**) Quantitative analysis of NeuN+ neurons cocultured for 40 div with NSC-derived astrocytes, differentiated for 21 div in areas with 25–30% GFAP+ cells over total DAPI+ nuclei. The reduction of neuronal cells is enhanced by high density of GFAP+ astroglial cells suggesting that neuronal demise is driven by cocultured astrocytes in a dose-dependent manner. (**b** and **e**) *t*-Test for all experiments was applied. Data are reported as mean±S.E.M.

We previously showed that an IDS deficit causes glial degeneration in mixed differentiated progeny from IDS-ko NSCs, which appeared before neuronal death.^2^ To assess the role of mutant astrocytes in neuronal degeneration, we cocultured NSC-derived pure astrocytes carrying the RFP (red fluorescent protein) reporter gene (lenti-rfp+ wt and IDS-ko astrocytes) with healthy rat primary cortical neurons. Wt and IDS-ko astrocytes were predifferentiated for 21 div to a postmitotic stage, and then plated on a culture of cortical neurons (20 000 astrocytes over 75 000 neurons), the latter containing very few astrocytes.^18^ The coculture was analyzed at 11, 20 and 40 div to assess the percentage of surviving neurons and the maintenance of functional neuronal markers (Figure 3). A time-course analysis of astroglial morphology over coculture showed that mutant astrocytes display a remarkable reactive morphology and tend to branch extensively through neuronal processes, whereas normal astrocytes look stellate and resident (Figure 3a). The number of astrocytes was similar during both wt and IDS-ko cocultures as no proliferation or significant cell death was detected by counting the relative number of astrocytes per unit of area. The expression of the dendritic marker MAP2 (microtubule-associated protein 2) was markedly reduced in neurons cocultured with mutant astrocytes compared with those cocultured with wt astrocytes, whereas the opposite trend was evident for astroglial rfp. Since confocal images of rfp+ cells were taken under same parameters and the intensity per area unit of IDS-ko cells was comparable to that of wt astrocytes, we assumed that rfp intensity correlates with cytoplasmic content, that is area of astrocytes (Figure 3b and c and Supplementary Figure 3a). Hence, neuronal degeneration occurred mainly in the coculture with mutant astrocytes. To test if the observed toxic effect on neuronal survival was dependent on astrocyte maturation, the cocultures were prepared with astrocytes predifferentiated for 7, 14 and 21 div. A significant neuronal degeneration became evident with astrocytes predifferentiated for 21 div (Figure 3d). In these cocultures, 17.1±3.4% of NeuN+ cells were detectable versus 34.9±4.6% in coculture with wt astrocytes (Figure 3d). Interestingly, these differences were enhanced in those areas of the coculture where mutant glia was more invasive and concentrated. Indeed, when we focused our quantitative analysis on areas with high astroglial density (with 25–30% of GFAP+ cells), the percentage of NeuN+ cells was 7.4±1.3% in coculture with IDS-ko astrocytes compared with 29.1±2.0% for wt (Figure 3e). No major neuronal death was observed in cultures of primary neurons alone. These results confirmed that IDS-ko astrocytes drive neuronal death in an age-dependent manner and likely through non-cell-autonomous effects.

As glial cells are known to be involved in synaptogenesis,^19^ we tested if mutant astrocytes can *per se* hamper synaptogenesis when cocultured with healthy neurons. We evaluated by immunofluorescence the expression of synapsin, a presynaptic protein specifically expressed by functionally active synapses. A reduction of synapsin spots was observed in healthy neurons when cocultured with mutant astrocytes at 20 div (Supplementary Figure 3a–c). Interestingly, this difference disappeared at 40 div, when sudden apoptosis and reduction of surviving neurons became remarkably evident (Supplementary Figure 3b). These results suggested that toxic effects mediated by mutant astrocytes might be involved also in controlling neuronal functioning or maturation, besides neuronal survival.

### Treatment with vitamin E rescues IDS-ko glial-mediated toxicity

**Figure 4:**
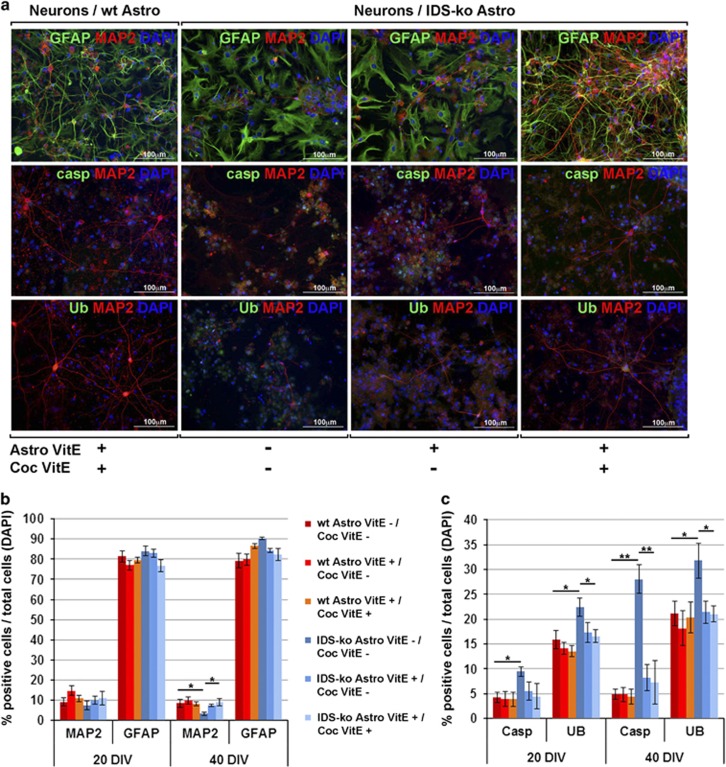
Treatment with vitamin E is able to rescue the IDS-ko glial-mediated toxicity on cocultured cortical neurons. (**a**) Panel of fluorescence microscopy images showing primary neurons (MAP2+, in red) cocultured with wt or IDS-ko astrocytes (GFAP+, in green) with or without treatment with vitamin E for 40 div and immunostained for Ub (green) and caspase-3 (green). Scale bars: 100 *μ*m. (**b**) Quantitative analysis of the number of astrocytes (GFAP+) and neurons (MAP2+) at 20 div and 40 div cocultured, differently treated with vitamin E. (**c**) Quantitative analysis of the number of caspase-3+ and Ub+ cells at 20 and 40 div cocultured, differently treated with vitamin E. One-way analysis of variance (ANOVA) for all experiments was applied. Data are reported as mean±S.E.M.

To show that a rescue of the mutant phenotype by vitamin E correlates with a rescue of the glial-mediated toxicity, we cocultured mutant astrocytes, previously predifferentiated in a vitamin E-enriched environment, with healthy neurons. The cocultures were carried on with or without the continuous administration of 10 *μ*M vitamin E^20^ for 40 div. No vitamin E toxicity was detected in the primary neurons (Supplementary Figures 4a and b). We observed that the survival rate of neurons cocultured with treated IDS-ko astrocytes was comparable to that of neurons cocultured with wt astrocytes. Both the fractions of GFAP+ and MAP2+ cells in treated cultures of mutant astrocytes were comparable to that of wt cells (Figures 4a and b), a result already evident at 20 div (Figure 4b and Supplementary Figure 4c). Importantly, comparable results were obtained by the quantitative analysis of caspase-3+ and Ub+ cells (Figure 4c and Supplementary Figure 4c), both colocalizing with either MAP2 (Figure 4a) or, to a major extent, with GFAP (Supplementary Figure 4c). However, a remarkable change of the morphological phenotype was seen at 40 div with continuous treatment with vitamin E: the shape of mutant astrocytes resembled that of the stellate wt astrocytes and a similar trend was observed for the branching and process elongation of neuronal cells (Figure 4a).

These results altogether showed that not only pretreatment of astrocytes with vitamin E is sufficient to reverse the mortality caused by the glial-mediated toxicity on cocultured neurons, but also that an uninterrupted treatment with vitamin E throughout the coculture appears able to trigger a full rescue of the phenotype, both of mutant glial and neuronal cells.

### Early neuroinflammation precedes glial degeneration in MPSII brain

**Figure 5:**
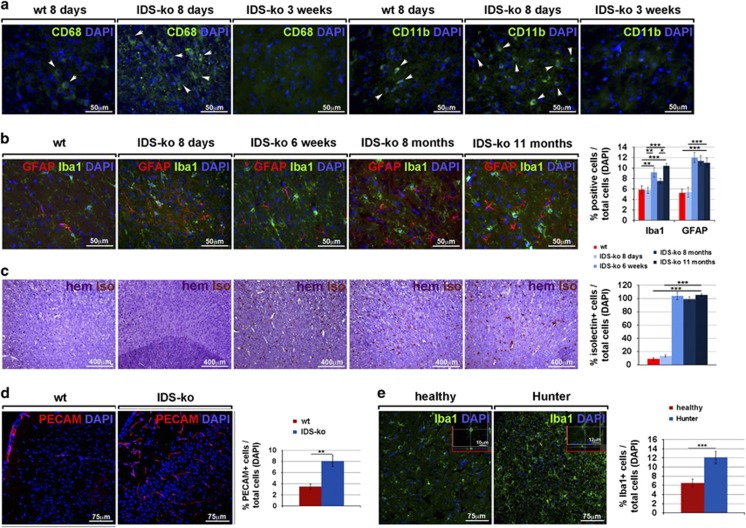
Neuroinflammation during MPSII progression. (**a** and **b**) Fluorescence microscopy images of wt and IDS-ko mouse brain cortex at different ages. The immunostaining of CD68, CD11b, Iba1 and GFAP, markers of macrophagic, microglial and astroglial cells, respectively, shows that the presence of acute inflammatory CD68+ and CD11b+ cells is evident in the early phase of the disease (**a**) and decreases from 3 to 6 weeks, whereas the following astrogliosis (GFAP+ cells) and microgliosis (Iba1+ cells) remain evident until the end stage (**b**). Quantitative analysis (graph) shows the increase of Iba1+ and GFAP+ cells in an age-dependent manner. Scale bars in (**a** and **b**): 50 *μ*m. (**c**) Histochemical staining with hematoxylin and isolectin, with related quantitative analysis, shows the increase of neovascularization occurring with aging in parallel with the development of a neuroinflammatory background. Scale bars: 400 *μ*m. (**d**) Confocal microscopy images of wt and IDS-ko mouse brain cortex at 8 months of age showing the immunostaining of the vascular marker PECAM. Scale bars: 75 *μ*m. The graph representing quantitative analysis of PECAM+ cells shows that a higher number of novel vessels is present in the mutant cortex compared with wt. (**e**) Confocal microscopy images of human cortex from healthy and Hunter patient showing immunostaining of Iba1 marker. Scale bars: 75 *μ*m. Scale bars in insets: 10–12 *μ*m. Quantitative analysis of Iba1+ cells shows the presence of active and massive microgliosis in the mutant compared with healthy brain at the end stage. One-way analysis of variance (ANOVA) was applied for (**b** and **c**), followed by Student's *t*-test for (**d** and **e**) experiments. All data are reported as mean±S.E.M.

The complex pathogenesis of LSDs probably include some systemic processes of non-neural cells, which cannot be recapitulated *in vitro* by pure NSC cultures. This is the case of neuroinflammation that we investigated in the IDS-ko mouse brain at different stages of the disease, looking for blood-infiltrating cells and microglial markers. In particular, we tested the expression of CD68 (cluster of differentiation 68 glycoprotein), expressed by infiltrating macrophages and endogenous microglia, and of CD11b (integrin *α*M), mostly expressed by monocytes, macrophages and microglia. Previous studies have shown that a transient increase of CD68+ and CD11b+ cells is present in the acute phase of neuroinflammation, due to a massive infiltration and migration of cells from the bloodstream to the site of injury. With the progression of neuroinflammation, the number of infiltrating cells from the bloodstream is dampened, whereas Iba1+ (ionized calcium-binding adapter molecule 1)-resident microglial cells change their morphology from a stellate to an amoeboid shape and increase in number until reaching a steady state at the chronic stage. We observed a significant number of CD68+ and CD11b+ cells in the cortex of IDS-ko brain at 8 days of age, whereas a remarkable decrease of positive cells was detected from 3 weeks of age onwards (Figure 5a). Interestingly, no difference in Iba1+ cells was evident at this stage, suggesting that the increase of CD68+ and CD11b+ cells was likely due to infiltrating cells; at 6 weeks of age, a higher number of Iba1+ cells with amoeboid-macrophagic morphology became evident in IDS-ko mice compared with wt mice, in which Iba1+ cells displayed a non-reactive stellate shape (Figure 5b and Supplementary Figure 5a). While macrophage activation appears to be enhanced (although not limited to) at the very early symptomatic phase of the disease, microgliosis increases with aging, and reaches a plateau at the symptomatic stage and is maintained at that level until the end stage (Figure 5a), with a terminal pattern similar to that observed in the brain of an advanced Hunter patient (Figure 5e).

Interestingly, in contrast to astroglial degeneration, limited to mature regions,^2^ microgliosis appeared as a global phenomenon detectable in both immature and mature brain areas (Supplementary Figure 5), in parallel with astrogliosis (Figure 5a).

As inflammation is related to oxidative damage and neovascularization of the pathological tissue in several conditions,^21^ we investigated the presence of novel blood vessels during disease progression in the IDS-ko mouse brain by isolectin immunohistochemistry. In the mutant brain, the percentage of isolectin+ cells was significantly higher compared with wt and increased with age (Figure 5c). Accordingly, immunofluorescence analysis of PECAM+ (platelet and endothelial cell adhesion molecule 1-positive) cells (Figure 5d) suggested that neovascularization could also participate, within the neuroinflammatory environment, in the development of murine MPSII pathogenesis.

### Early white matter degeneration in Hunter patients

**Figure 6:**
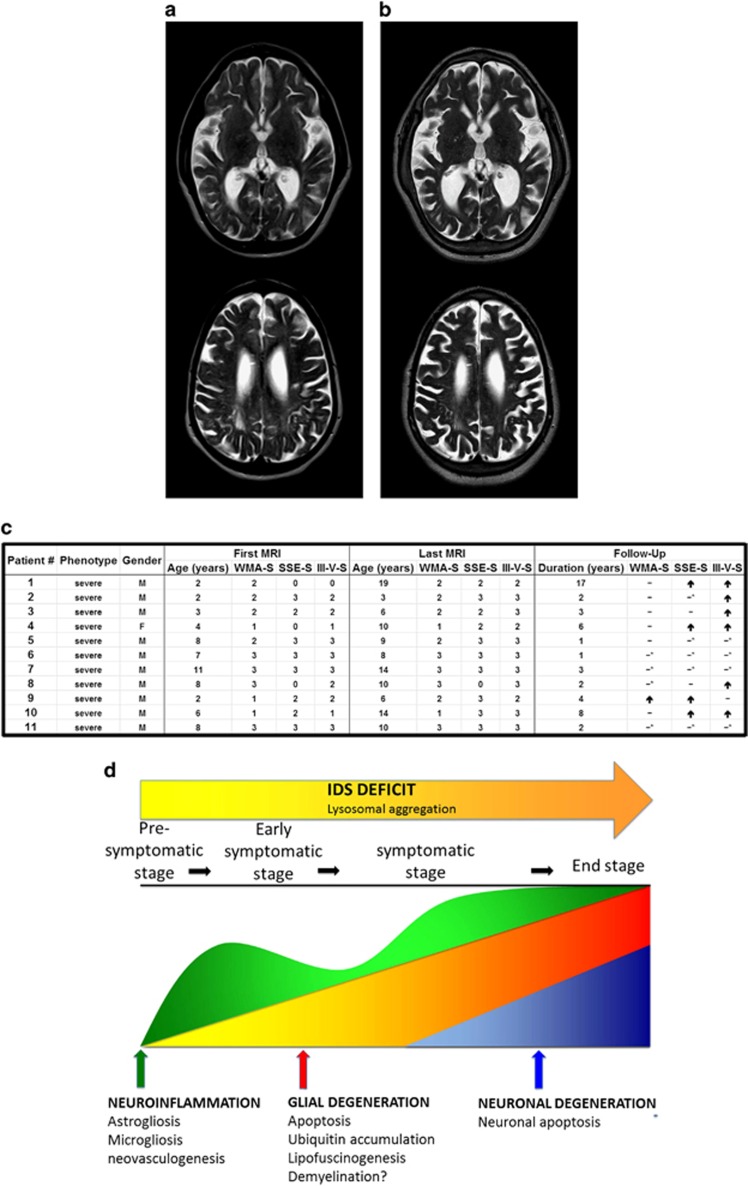
White matter degeneration precedes neuronal dysfunction in Hunter's patients. (**a** and **b**) Brain MRI of an MPSII patient with severe phenotype. Axial T2-weighted images at the level of the basal ganglia (upper row) and at the level of the upper part of the lateral ventricles (lower row). a) At the age of 8 years, periventricular white matter signal abnormalities and severe enlargement of the cerebrospinal fluid spaces (ventricles and subarachnoid spaces) are evident. (**b**) At the age of 12 years, the atrophy has progressed with further enlargement of the ventricles and the sulci of the convexity, whereas no significant worsening of the white matter was recognizable. (**c**) Table showing the progression of alterations during MPSII disease development in 11 severe patients. (**d**) Model of MPSII disease development and progression according to mainly three stages: IDS deficit causing GAG accumulation with immunogenic response at the immediate postnatal stage concurs with oxidative damage and impairment of mitochondrial pattern to trigger a massive neuroinflammation (stage 1), consisting of simultaneous microgliosis, astrogliosis and neovascularization. The early development of an inflammatory background together with inherent IDS deficit leads to progressive astroglial degeneration (stage 2) that finally drives neuronal death in an age-dependent manner (stage 3). M, male; F, female; SSE-S, subarachnoid space enlargement score; III-V-S, third ventricle enlargement score; ↑, worsened; −, unchanged; −*, highest score at first examination; WMA-S, white matter abnormality score

In the context of a routine clinical follow-up, 11 MPSII patients with severe phenotype were repeatedly evaluated through brain magnetic resonance imaging (MRI) for white matter signal abnormalities (WMAs) and atrophy (ventricle and subarachnoid space enlargement) according to previously reported scores.^22^ All patients exhibited WMAs at the first MRI (mean score 2.1, range 1–3); one patient showed a worsening WMA during follow-up. Ten patients had signs of brain atrophy at first MRI (mean score 2.0 for both ventricle and subarachnoid space enlargement range 0–3). Six patients showed progressive ventricle enlargement during the follow-up, 4/5 of the remaining patients reached the maximum score already at first examination. Similarly, four patients presented progressive subarachnoid space enlargement, whereas 5/7 of the remaining patients had already the maximum score at first examination. Therefore, *in vivo* MRI findings confirmed that the white matter involvement mostly occurs during the very first years of age in severe MPSII patients and shows limited evolution during subsequent follow-up (Figures 6a and b). In contrast, while signs of atrophy are mostly present already at diagnosis, the neurodegenerative process seems to endure thoroughly during the follow-up (Figure 6c). These finding is consistent with our previous analysis *in vitro* and in IDS-ko mouse brain showing glial before neuronal atrophy.

As a whole, our study suggests a novel model of MPSII pathogenesis (Figure 6d), with neuroinflammation preceding glial degeneration, which is finally followed by neuronal death.

## Discussion

We recently showed that glial degeneration in MPSII precedes neuronal damage both in NSCs derived from the IDS-ko mice and in the brain, obtained from the same model and from a Hunter patient.^2^ In the present work, we confirm that degeneration of glial cells concurs with neuroinflammation to foster neuronal death and show that oxidative stress is one of the candidate mechanisms involved in MPSII pathogenesis.

We first characterized the pure astrocyte culture obtained from IDS-ko NCSs and observed that increased lysosome number and size, a hallmark of MPSII, is particularly evident in mature astrocytes compared with standard differentiated NSCs, confirming that isolated astrocytes are remarkably affected by IDS deficit, and that pathogenic hallmarks increase with long-term differentiation.

Given the reported involvement of mitochondria and lysosomes and of their crosstalk in apoptosis,^8^ we analyzed the mitochondrial distribution. An anomalous mitochondrial pattern was remarkable in astrocytes, supporting the presence of oxidative damage in MPSII progression.^23^ Similar analysis in postmitotic fibroblasts from Hunter patients showed that mitochondrial alterations are also present in humans. As lipofuscinogenesis is one of the important manifestations of ROS-induced damage occurring within the lysosomal compartment,^8^ and mitochondria are a major source of the macromolecules from which lipofuscin forms,^8^ we tested the presence of lipofuscin in IDS-ko NSC-derived astrocytes and in IDS-ko mouse brain. In support of previous studies showing a significant storage only in long-lived non-dividing cells,^8^ lipofuscin was remarkably evident in mutant astrocytes in an age-dependent manner both *in vitro* and *in vivo*. This finding suggested that oxidative stress may act upstream of and promote glial degeneration. Indeed, we observed that culturing IDS-ko NSCs under low oxygen conditions or in the presence of vitamin E throughout differentiation induces a reduction of lysosomal alterations and lipofuscinogenesis and an ensuing decrease of apoptotic cells. Here we show that antioxidant treatment may limit damage in IDS-ko astrocytes. Thus, the exploitation of antioxidant drugs could supplement the previously reported gliotrophic molecules such as platelet-derived growth factor^2^ in preventive care aimed at delaying the disease onset and slowing its progression. Prior work of others^5^ reported an excess of markers of oxidative stress and a reduction of non-enzymatic antioxidants in Hunter patients.

We previously observed that lysosomal alterations in IDS-ko GFAP+ cells are age-dependent both *in vitro* and *in vivo.*^2^ The observed changes in the differentiation capability of IDS-ko NSCs, both to mixed neural progeny^2^ and, in this study, to mature astrocytes, correlate with the developmental process of Hunter CNS, in that glial cells degenerate from the early stages of the disease, whereas neuronal cells mostly die from the late symptomatic to the end stage.^2^

To mimic the pathogenic context *in vivo* and to determine if IDS-ko astrocytes could exert a toxic effect on neurons independently of an IDS deficit in these cells, we cocultured IDS-ko astrocytes with rat primary neurons, endowed with earlier capacity to mature and longer survival ability compared with mouse primary neurons.^24^ We observed a reduction of the synaptic density, and a progressive apoptosis accelerated by the presence of IDS-ko astrocytes, suggesting that these cells are able *per se* to compromise neuronal function and to drive neuronal degeneration through non-cell-autonomous effects. Most importantly, we showed that early treatment of differentiating mutant astrocytes with vitamin E could rescue the glial-mediated toxicity on cocultured neuronal cells and that the uninterrupted administration of vitamin E mostly rescues the wt phenotype both in IDS-ko astrocytes and in cocultured neurons.

This evidence suggests that oxidative damage may be the primary event leading IDS-ko astroglial cells to drive both neuronal death and differentiation impairment. Thus, a very early and noninvasive antioxidant therapy could potentially delay MPSII progression.

After observing the development of a neuroinflammatory phenotype in IDS-ko-differentiating NSCs and in IDS-ko mouse brain with aging, we investigated the temporal pipeline of neuroinflammation along MPSII pathogenesis *in vivo*. Incipient macrophagic infiltration is clearly evident in the MPSII brain at the postnatal stage with subsequent microgliosis, astrogliosis and parallel enhancement of neovascularization, which is associated with oxidative stress and inflammation.^21^ The studies from IDS-ko mice are supported by immunofluorescence analysis of an autopsy specimen from a unique Hunter patient's brain^2^ (Figure 5e) and by MRI analysis of multiple Hunter patients' brains. These results support a three-stage model of MPSII pathogenesis: primary neuroinflammation (stage 1) that is already evident at the postnatal stage together with neovascularization and oxidative damage (Figure 6d); these initial events, together with intrinsic metabolic impairment due to IDS deficit, drive the subsequent glial degeneration (stage 2), which finally leads to neuronal demise (stage 3). Although beyond the aim of this study, the specific mechanisms involved in neuroinflammation's onset and progression and the correlation of microgliosis/astrogliosis with the following glial apoptosis *in vivo* would deserve further investigation.

Our findings together indicate that multiple mechanisms act early in MPSII pathogenesis. These studies also suggest that antioxidant drugs may be candidates to delay onset and disease progression in the developing MPSII brain, and complement enzyme replacement in addressing the oxidative stress that has been documented previously in MPSII patients.^5, 6^ The evidence of oxidative stress early in pathogenesis makes early diagnosis in severe MPSII patients of the utmost importance.^25^

## Materials and Methods

### Mice

C57BL6 IDS-ko mice were kindly provided by J Muenzer (University of North Carolina, Chapel Hill, NC, USA), C57BL6 wt mice were purchased from Harlan Italy (Milano, Italy). IDS-ko and wt mice from the same littermate were used. Mice were housed in light- and temperature-controlled conditions, with food and water provided *ad libitum*. All animal care and experimental procedures were conducted according to the current national and international animal ethics guidelines.

### Human specimen

Human brain tissue was obtained from the NICHD Brain and Tissue Bank for Developmental Disorders at the University of Maryland (Baltimore, MD, USA).

### Human cells

Human fibroblasts from skin biopsy of three Hunter pediatric patients were kindly obtained from the ‘Cell Line and DNA Bank from Patients Affected by Genetic Diseases' (a Telethon Genetic Biobank) (Gaslini Institute, Genova, Italy). As healthy controls, human fibroblasts from three circumcisions were used; they were obtained from the Histology Unit of the Department of Histology, Microbiology and Medical Biotechnology (University of Padova, Padova, Italy). All cells were anonymously obtained. Primary fibroblasts were cultured at 37 °C in 5% CO_2_ atmosphere in RPMI medium supplemented with 15% fetal bovine serum (FBS), 100 U/ml penicillin and 100 ng/ml streptomycin (Life Technologies, Carlsbad, CA, USA).

### Ethics statement

Animals were housed at the University of Padova under the supervision of the ‘Comitato Etico per la Sperimentazione Animale' of the University of Padova. For the other practices, about removal of organs, all animal care and experimental procedures were conducted according to the current national and international animal ethics guidelines of the Università Milan Bicocca (Milano, Italy). All the experimental protocols were approved by ‘Comitato Etico per la Sperimentazione Animale' of the University of Padova or by Comitato Etico of University Milano-Bicocca.

Human fibroblasts from skin biopsy of three Hunter pediatric patients were obtained from the ‘Cell Line and DNA Bank from Patients Affected by Genetic Diseases' (Gaslini Institute).

### Propagation of mouse NSCs

Two wt and two IDS-ko NSC lines were isolated and propagated from the SVZ of adult mouse brains at 6–7 weeks of age as described elsewhere.^2^

Here all results will be presented as the average of two IDS-ko and of two wt NSC lines, respectively.

### Differentiation of NSCs

To induce NSC differentiation, individual spheres were mechanically dissociated and cells were transferred at 2.5 × 10^4^ cells per cm^2^ onto cultrex-coated chamber slides and differentiated as described in Gritti *et al.*^26^ for a total of 10 days.

For differentiation to pure astrocytes, neurospheres were propagated for two passages in basal medium containing fibroblast growth factor-2 (Peprotech, Rocky Hill, NJ, USA) and FBS 2% and then terminally differentiated for 21 div in basal medium containing FBS 10% at a density of 20 000 astrocytes per cm^2^.

Where indicated, cells were incubated throughout differentiation under standard (16–20% O_2_) or low (5%) oxygen condition or treated with 10 *μ*M vitamin E (Sigma Aldrich, Darmstadt, Germany).^20^

### Immunocytochemistry

Cultures were fixed in freshly prepared, buffered 4% paraformaldehyde. After blocking with 10% normal goat serum (NGS), cultures were incubated overnight at 4 °C with the following antibodies (mAbs, monoclonal; pAbs, polyclonal): *β*-tubulin isotypeIII (*β*-tubIII, mAbs, MMS-435 P (Covance, Princeton, NJ, USA), 1 : 400), GFAP (pAbs (Dako, Cernusco sul Naviglio, Milan, Italy), 1 : 400), Lamp1 (pAbs, ab24170 (Abcam, Cambridge, UK), 1 : 750), cleaved caspase-3 (Casp; Asp 170) (pAbs, no. 9961 (Cell Signaling Technology, Danvers MA, USA), 1 : 500), Ub (pAbs, Z0458 (Dako), 1 : 50) MAP2 (mAbs (Chemicon, EMD Millipore, Darmstadt, Germany), 1 : 400).

After removal of the primary Abs and repeated washes with Dulbecco's phosphate-buffered saline (PBS), cultures were incubated at room temperature for 45 min with secondary antibodies labeled with Alexa Fluor 594 or 488 (anti-mouse and/or anti rabbit; Molecular Probes, ThermoFisher Scientific, Waltham, MA, USA). Samples were then colored with DAPI (4′,6-diamidino-2-phenylindole; 0.3 *μ*g/ml; Roche, Basel, Switzerland) for nuclear staining and rinsed with PBS for mounting and analysis. Microphotographs were taken using a Zeiss Axiovert 200 direct epifluorescence microscope (Axioplan 2; Carl Zeiss, Jena, Germany) or a confocal microscopy (Leica DM IRE2, Milan, Italy).

### Immunohistochemistry

Mice (from 8 days to 11 months old, as indicated in the text) were killed with Avertin (300 mg/kg) and transcardially perfused fixed with 4% paraformaldehyde. Brains were postfixed overnight, cryoprotected, frozen and coronally sectioned (20 *μ*m thick) by cryostat. For the human specimen, sections (20 *μ*m thick) were obtained by cryostat, postfixed with 4% paraformaldehyde and processed as murine samples. Sections were blocked with 10% NGS and 1% Triton X-100 for 90 min and incubated overnight with the following primary antibodies: GFAP, *β*-tubIII, MAP2, Lamp1, Casp, Ub Iba1, CD68 and CD11b (Abbiotec, BBIO, San Diego, CA, USA). The fluorescent secondary antibodies used were labeled with Alexa Fluor 549 or 488 (Molecular Probes). DAPI (see immunocytochemistry analysis) was used as the nuclear marker. Labeled samples were analyzed using a fluorescence microscopy and a confocal microscopy (see immunocytochemistry analysis).

### Coculture of NSC-derived astrocytes with primary cortical neurons

NSC-derived astrocytes were obtained as described above.

Primary rat cultures were extracted from 18- to 19-day-old rat embryos (pregnant female rats were obtained from Charles River Laboratories, Wilmington, MA, USA). Neurons were plated at low density (75–80 cells per mm^2^) and grown as described previously.^27^ At 1 div astrocytes, previously infected (where indicated) with lentiviral vector carrying RFP reporter gene as described in Follenzi *et al.*,^28^ were detached with Versene, collected and plated onto the cortical neurons (20 000 astrocytes/75 000 neurons). The coculture was maintained with or without 10 *μ*M vitamin E and analyzed by immunocytochemistry at 11, 20 and 40 div.

### Lipofuscin and Nissl staining

Lipofuscin and Nissl staining were performed as described previously.^29^

### Isolectin histochemical analysis

Isolectin histochemical analysis was performed as described previously.^30^

### JC1 mitochondrial assay

Mitochondrial membrane staining was evaluated with the lipophilic cationic probe JC-1 (Invitrogen, Carlsbad, CA, USA). Cells were stained with 0.5 *μ*M JC-1 and kept at 37 °C for 15 min (fibroblasts) and for 20 min (neural cells), washed with PBS and analyzed by a fluorescence microscope.

### MitoTracker assay

Human fibroblasts were stained with MitoTracker Red CMXRos (Life Technologies, Carlsbad, CA, USA) 300 nM for 30 min, and then fixed with 3.7% formaldehyde, according to the manufacturer's protocol. Micrographs were taken by confocal microscopy.

### Image quantification and statistical analysis

For immunocytochemistry, data are reported as percentages of labeled cells over the total number of nuclei±S.E.M. An average of 3 × 10^3^ total cells (identified by DAPI nuclear staining) was counted randomly from two coverslips per condition in each experiment. Each value represents the average of three independent experiments.

For immunohistochemistry, quantification of the percentage of GFAP+ cells over total DAPI was performed in three regions of interest per section (CTX, cortex; SVZ/STR, subventricular zone (each 200 *μ*m apart) spanning the central region of bregma 0 and adjacent striatum area; OB, olfactory bulbs). Each value represents the average of *n*=3 animals unless differently stated in the text.

For the Mitotracker assay, mitochondrial chain length was estimated by manually tracing unbranched mitochondria on confocal images and measuring their length using the ImageJ software (U.S. National Institutes of Health, Bethesda, MD, USA). Counts were performed in 14 cells per patient, with a mean of about 33 isolated mitochondrial chains counted per cell.

Statistical analysis was performed by Student's *t*-test and one-way ANOVA (Bonferroni test). Data are reported as mean±S.E.M. and are considered not statistically significant unless indicated in the figures (*P⩽0.05, **P⩽0.01, ***P⩽0.001).

### Western blot analysis

Immunoblots were performed as described in Carlessi *et al.*^31^ Membranes were incubated with rabbit antibodies against Lamp1 (Cell Signaling Technology) and mAbs for tubulin (Sigma), the latter to normalize bands for equal loading of proteins per lane. Bands were quantified by densitometric analysis of the ECL-exposed films.

### Brain MRI

Eleven MPSII patients (10 males and 1 female) with severe phenotype underwent repeated brain MRI examinations, in the context of a routine clinical follow-up. The mean age at first and last MRI were 5.5 and 9.0 years with ranges 2–11 and 3–19, respectively; the mean follow-up lasted 4.4 years with a range of 1–17 years. WMAs and atrophy, third ventricle enlargement (III-V) and subarachnoid space enlargement (SSE) were evaluated according to previously reported scores.^22^

## Abbreviations

Astro: pure astrocytes
*β* -tubIII: *β*-tubulin isotypeIII
Casp: cleaved caspase-3
cc: corpus callosum
CD11b: integrin *α*M
CD68: cluster of differentiation 68 glycoprotein
CNS: central nervous system
CTX: cortex
DAPI: 4′,6-diamidino-2-phenylindole
DIV: days *in vitro*
ERT: enzyme replacement therapy
FBS: fetal bovine serum
GAG: glycosaminoglycan
GFAP: glial fibrillary acidic protein
IBA1: ionized calcium-binding adapter molecule 1
IDS: iduronate 2-sulfatase
Lamp1: lysosomal-associated membrane protein 1
LSD: lysosomal storage disease
MAP2: microtubule-associated protein 2
MPSII: mucopolysaccharidosis type II
MRI: magnetic resonance imaging
NSC: neural stem cell
PBS: Dulbecco's phosphate-buffered saline
PECAM: platelet and endothelial cell adhesion molecule 1
RFP: red fluorescent protein
ROS: reactive oxygen species
S.E.M.: standard error of the mean
SSE-S: subarachnoid space enlargement score
SVZ: subventricular zone
Ub: ubiquitin
WMA: white matter signal abnormality
WMA-S: white matter abnormality score
wt: wild type
III-V-S: third ventricle enlargement score
