# Supplementary material for: Glial degeneration with oxidative damage drives neuronal demise in MPSII disease
Source: Cell Death Dis. 2016 Aug 11;7(8):e2331–. doi: 10.1038/cddis.2016.231 (PMC5108318; doi:10.1038/cddis.2016.231)
Supplement: Supplementary Figure Legends [file cddis2016231x1.docx]

**Supplementary Figure 1. Lipofuscinogenesis in murine and human IDS-ko astrocytes**

**a)** Nissl staining of IDS-ko mouse brain cortex shows the presence of important neuronal degeneration at 11 months of age. Quantitative analysis of surviving neurons over total nuclei shows that neuronal demise is particularly evident in the cortical area compared to SVZ. The differences among all the values were statistically not significant unless indicated (*p≤0.05, ** p≤0.01, *** p≤0.001); one-way ANOVA was applied. **b-c)** Thionin histochemical staining of lipofuscin storages (red arrows) in both wt and IDS-ko mixed differentiated NSCs or astrocytes (b, top), wt and IDS-ko mouse brain cortex (b, bottom) and human Hunter’s brain (c) show a remarkable lipofuscinogenesis in mutant astrocytes (insets) compared to those wt. Scale bars a-b: 50μm. Scale bars in b insets: 15-20μm. Scale bars c: 7-10μm.

**Supplementary Figure 2. Vitamin E mimics low oxygen effects on lipofuscinogenesis and glial degeneration**

**a)** Wt and IDS-ko NSC derived astrocytes were cultured for 21 div with or without (control) the presence of Vitamin E in the culture medium. Phase contrast images show the major spreading of the cell body and processes. Scale bars: 75μm. Immunostaining with antibodies against Lamp1, ubiquitin (Ub) and caspase 3 (casp) show the reduced number of lysosomal aggregates and of apoptotic cells in mutant astrocytes treated with Vitamin E compared to control. Scale bars: 50μm. **b)** The graph represents the quantitative analysis of caspase3+ and ubiquitin+ cells over the total DAPI+ nuclei under standard and low oxygen conditions. The differences among all the values were statistically not significant unless indicated (* p≤0.05, ** p≤0.01, *** p≤0.001); Student’s t-test for all experiments was applied. **c)** Thionin histochemical staining of lipofuscin storages in IDS-ko astrocytes under 5% O_2_ condition and treated with Vitamin E show a remarkable reduction of lipofuscinogenesis compared to control (20%O_2_ untreated). Scale bars: 100μm. Scale bars in insets: 20μm.

**Supplementary Figure 3. Effects of mutant astrocytes on synaptic machinery of healthy neurons**

**a)** Confocal microscopy images of cocultures of primary neurons and NSC derived wt/mutant astrocytes (21 div) immunostained with the pre-synaptic marker synapsin show that neuronal death conditioned by mutant astrocytes impair synaptic density. Scale bars: 75μm.

**b-c)** graphs representing the quantitative analysis of synapsin normalized over MAP2 signal intensity **(b)** and of synapsin+ punctate buttons normalized over the relative neuronal process length **(c)** show a reduction of synapsin expression in neuronal processes from 20 div of co-culture with mutant astrocytes that is followed by massive neuronal death. The differences among all the values were statistically not significant unless indicated (* p≤0.05, ** p≤0.01, *** p≤0.001); Student’s t-test for all experiments was applied.

**Supplementary Figure 4. Treatment with Vitamin E is not toxic to primary neurons and rescues IDS-ko glial-mediated toxicity on co-cultured neurons already at 20 div**

**a-b)** Fluorescence microscopy images (a) and quantitative analysis (b) of primary neurons treated with or without 10μM Vitamin E and immunostained with MAP2 antibody. **c)** Panel of fluorescence microscopy images showing primary neurons (MAP2+, in red) co-cultured with wt or IDS-ko astrocytes (GFAP+, in green) with or without treatment with Vitamin E for 20 div and immunostained for ubiquitin (green) and Caspase3 (green). Scale bar: 100µm. The differences among all the values were statistically not significant (Student’s t-test for all experiments was applied).

**Supplementary Figure 5. Neuroinflammation is diffused throughout the Hunter brain**

**a)** Confocal microscopy images of wt and IDS-ko mouse brain immunostained for the microglial marker Iba1 (b). The presence of microgliosis is evident in striatum, cortex (CTX) and olfactory bulbs of IDS-ko brain compared with wt sibling. The morphology of microglial Iba1+ cells in IDS-ko brain is macrophagic-amoeboid with respect to the stellate resident microglia in control brains (insets). Scale bars: 75-100 μm; in insets: 10-12 μm. **b)** Quantification of Iba1+ cells over total DAPI+ nuclei confirms a remarkable microgliosis in all the brain areas analyzed. The differences among all the values were statistically not significant unless indicated (*p≤0.05, n.s., ** p≤0.01, ***, p≤0.001); one-way ANOVA followed by the Student’s t-test was applied for all experiments.
