# Supplementary figures and images for: Glial degeneration with oxidative damage drives neuronal demise in MPSII disease
Source: Cell Death Dis. 2016 Aug 11;7(8):e2331–. doi: 10.1038/cddis.2016.231 (PMC5108318; doi:10.1038/cddis.2016.231)

Figure S1

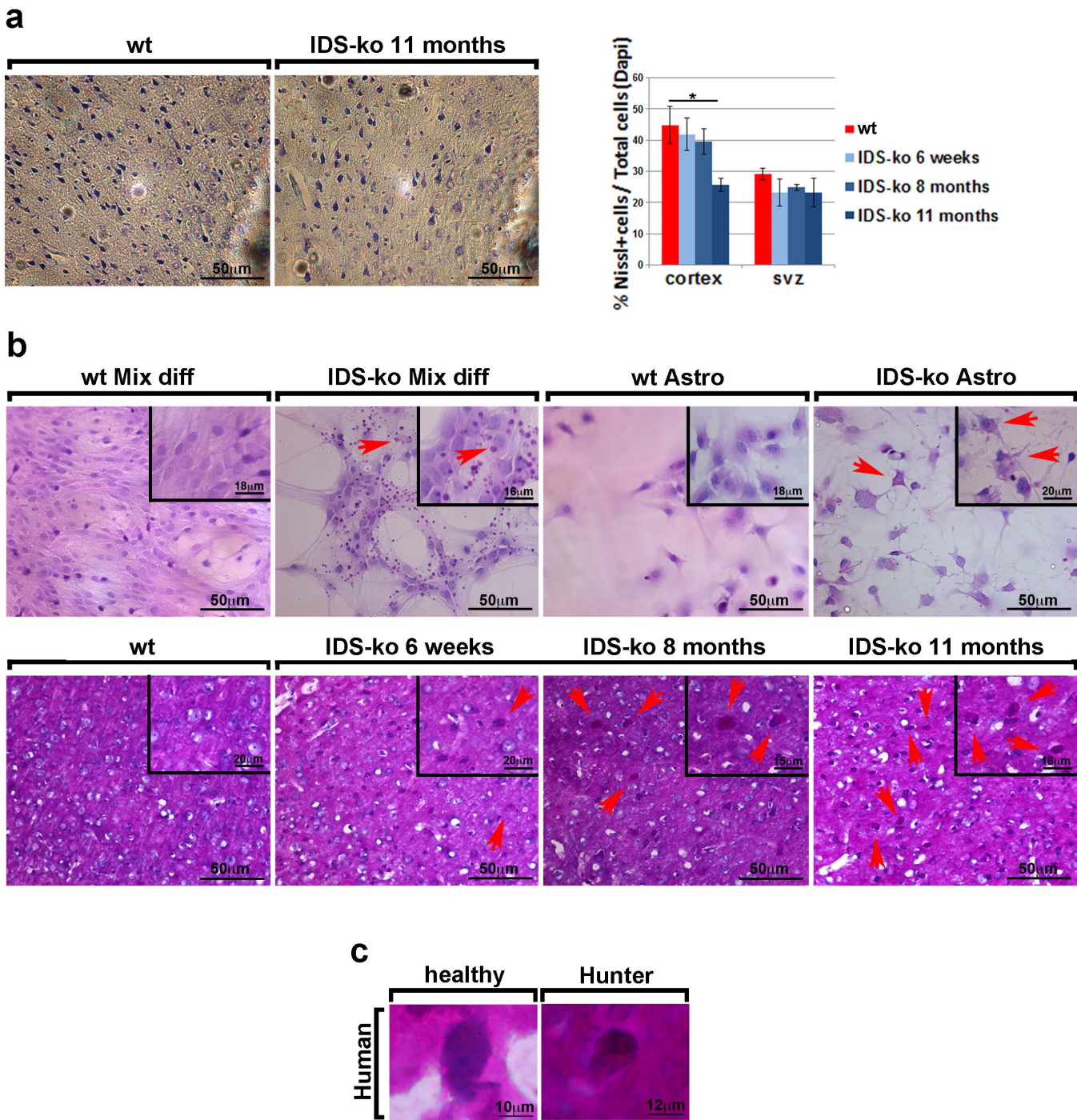

Figure S2

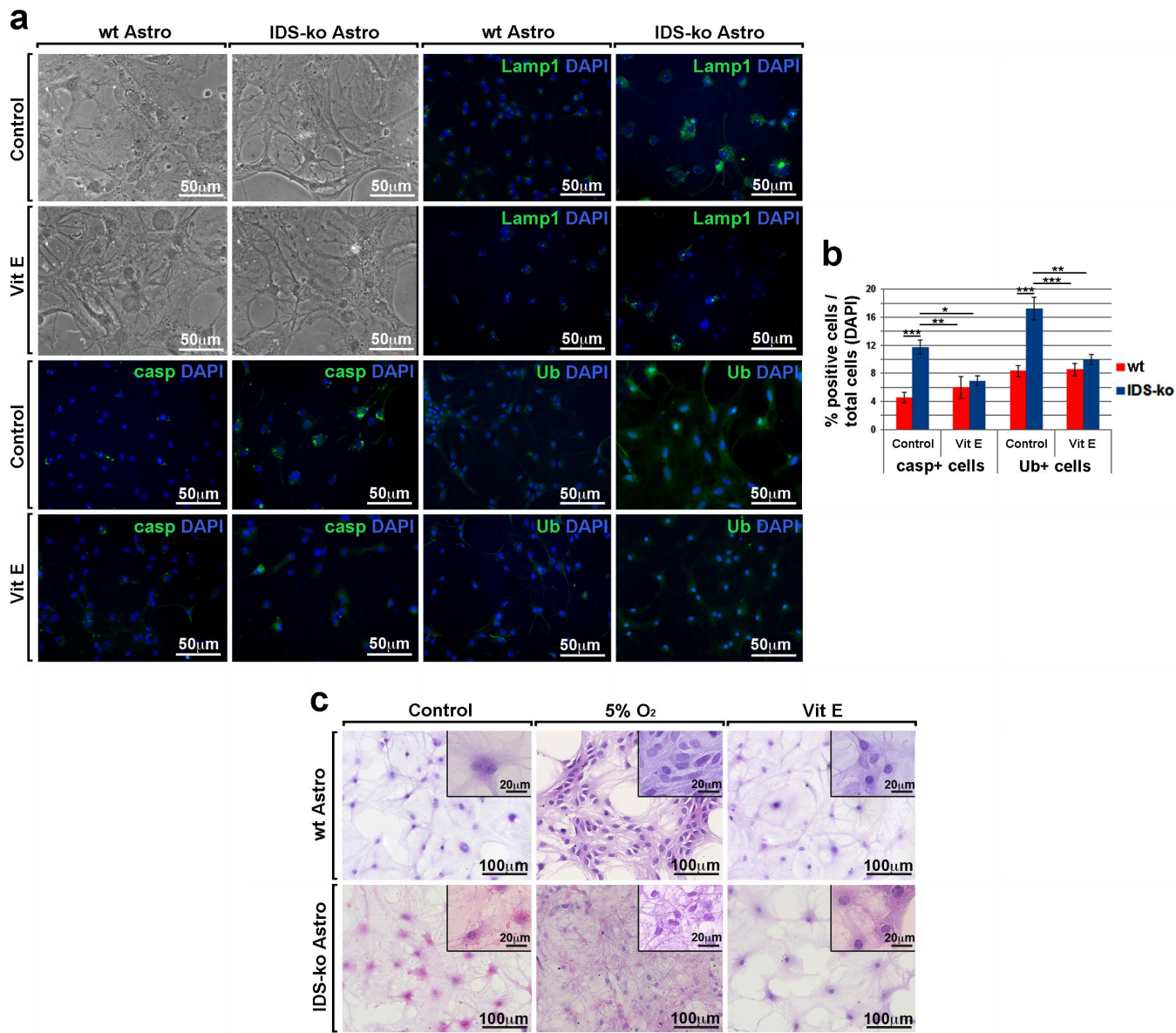

Figure S3

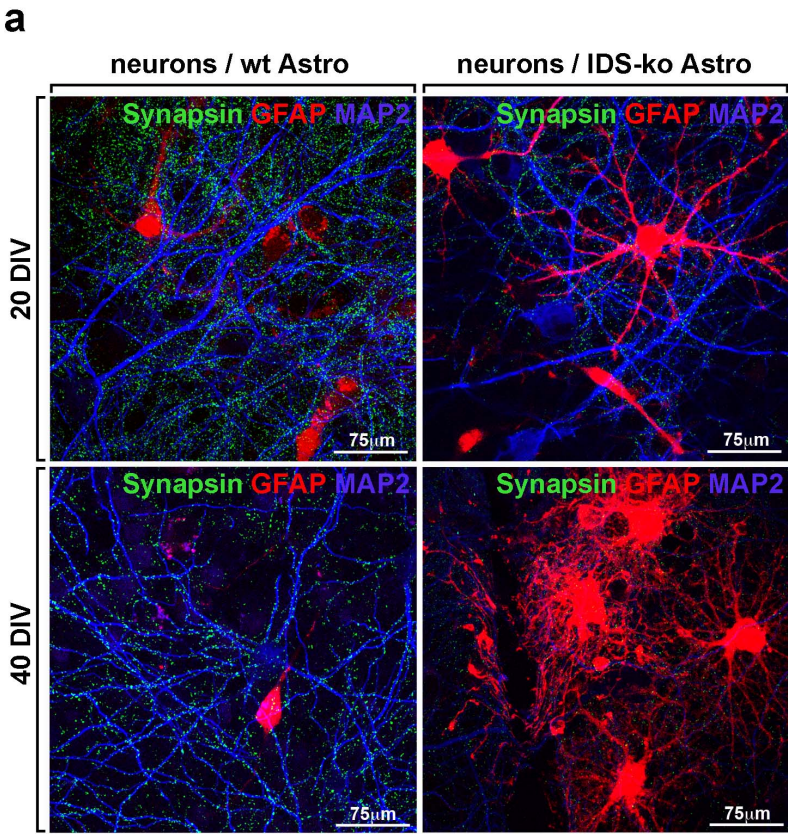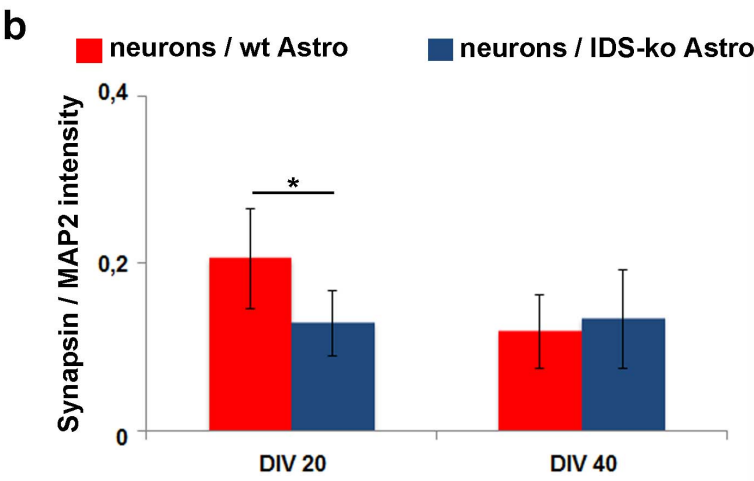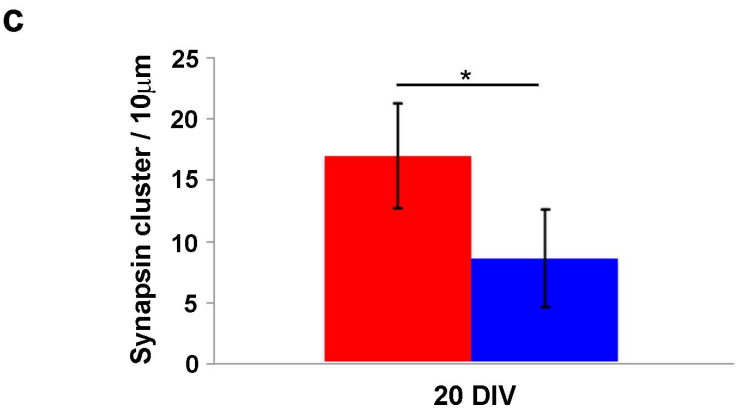

Figure S4

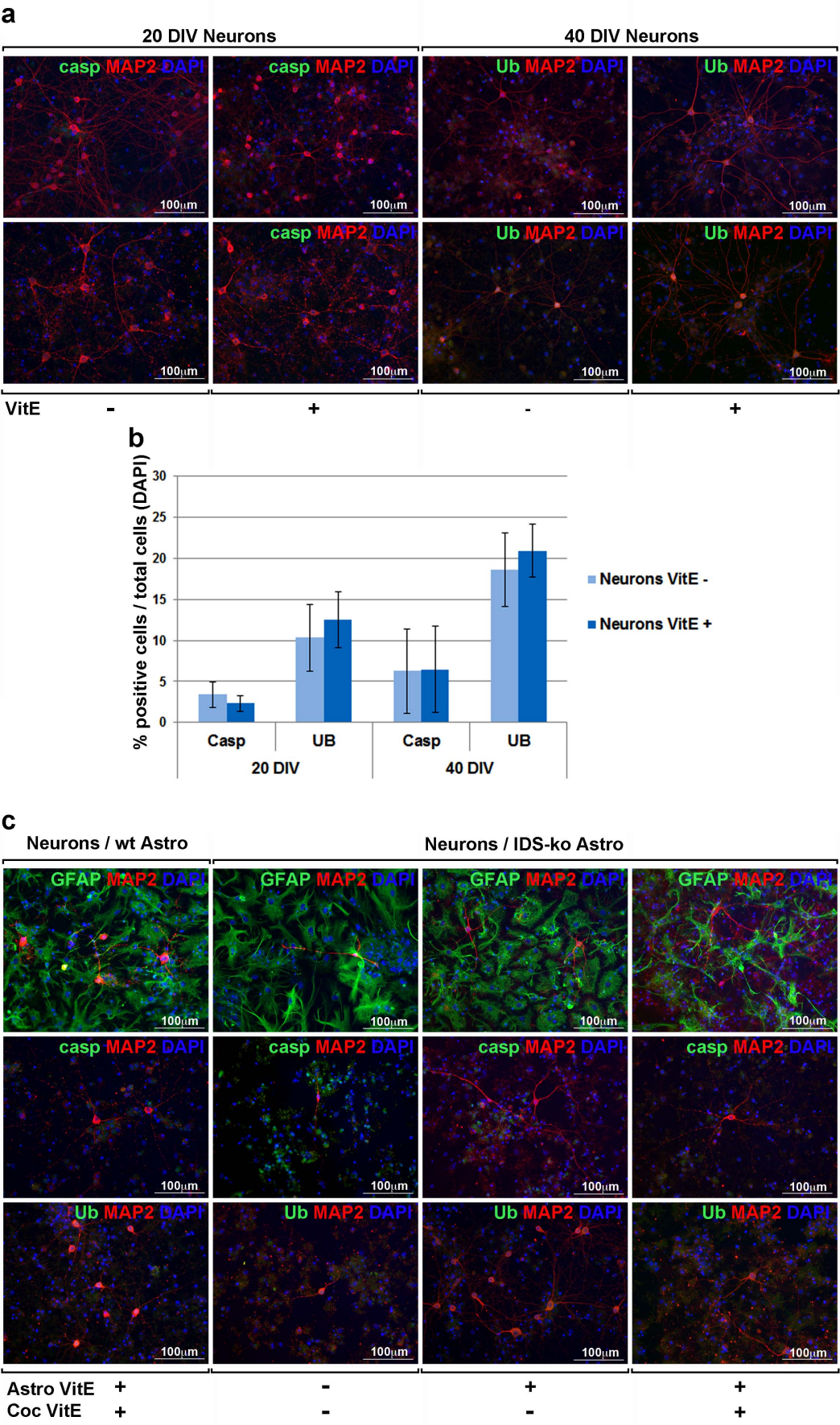

Figure S5

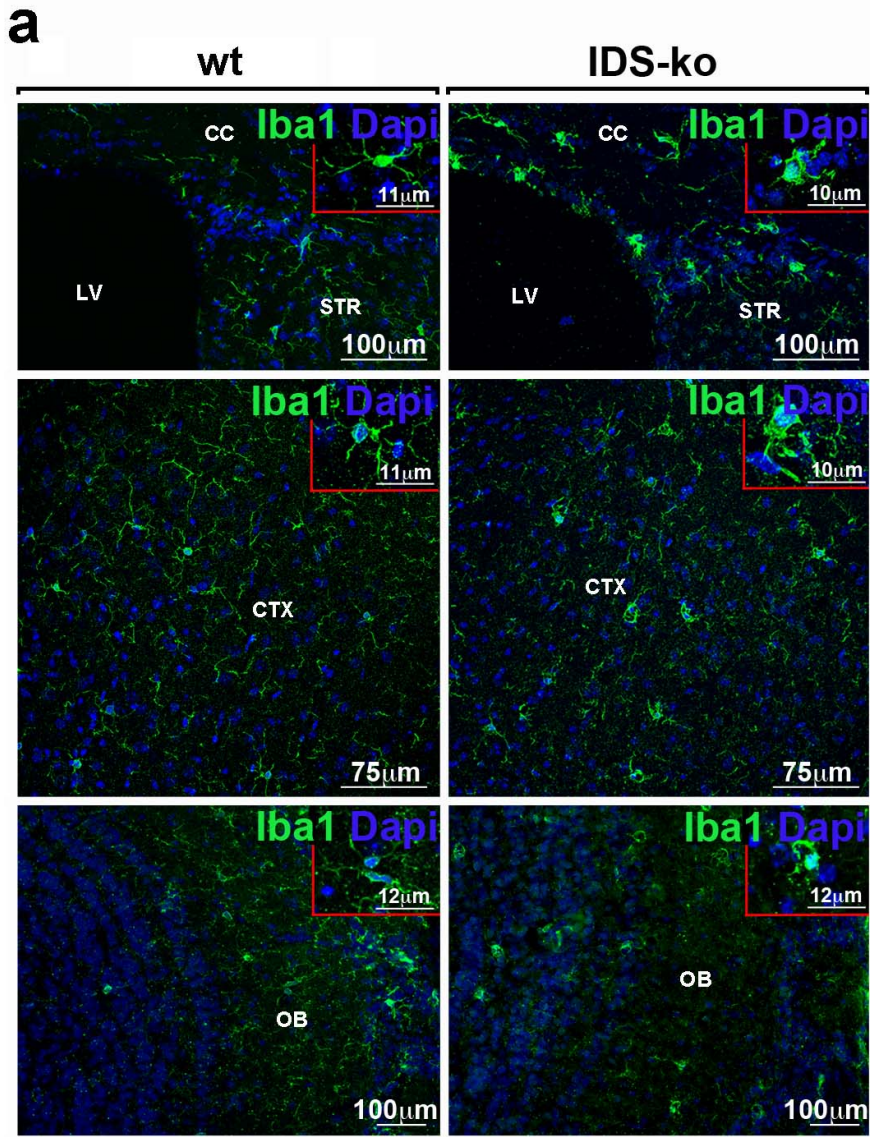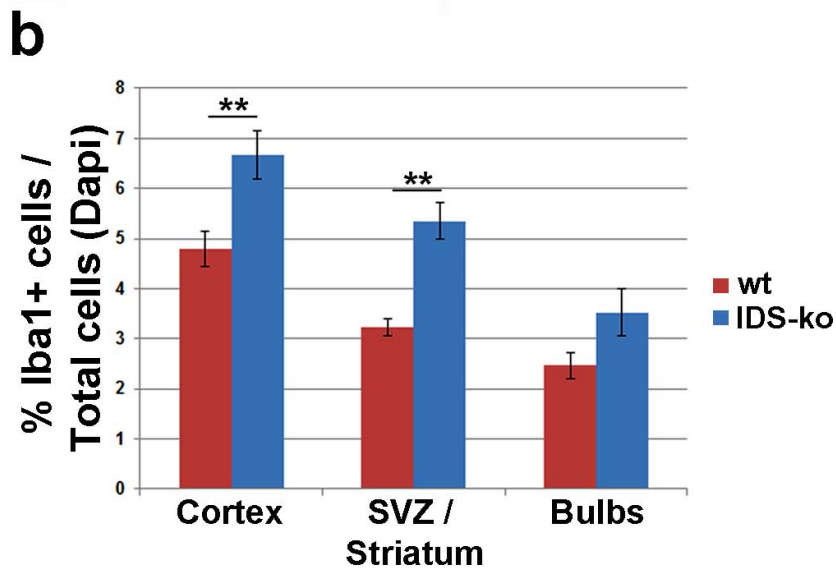

Supplement: Supplementary Figures [file cddis2016231x2.pdf]
